# Placental weights of neonates born with symptomatic congenital syphilis

**DOI:** 10.3389/fped.2023.1215387

**Published:** 2023-10-06

**Authors:** Shakti Pillay, Alan R. Horn, Lloyd Tooke

**Affiliations:** ^1^ Neonatal Unit, Groote Schuur Hospital, Cape Town, South Africa; ^2^ Division of Neonatology, Department of Paediatrics, University of Cape Town, Cape Town, South Africa

**Keywords:** placenta, syphilis, congenital, heavy, infection

## Abstract

**Background:**

Syphilis during pregnancy remains an important global health concern causing miscarriage, stillbirth, preterm birth and neonatal death. As part of the fetal infection, placental changes occur which may include a heavier placenta than expected.

**Methods:**

A cohort of 50 neonates with symptomatic congenital syphilis has previously been described. This cohort was admitted to Groote Schuur neonatal unit in Cape Town South Africa from 2011 to 2013. For this study, the placental weights of the neonates were analyzed and compared to population based placental centiles.

**Results:**

There was data for 37 placentae. Heavy placentae (>90th centile) occurred in 76% of placentae in the study. All 6 infants with birth weights ≥2,500 g had heavy placentae. There was no correlation between placental centile and death.

**Conclusion:**

Heavy placenta are an important and frequent finding with symptomatic congenital syphilis, especially in the larger neonates.

## Introduction

Despite effective antenatal screening and treatment, syphilis remains one of the commonest congenital infections globally and a serious public health concern (1, 2). In 2016, the World Health Organisation estimated the global prevalence of syphilis among pregnant woman to be 0.69%. Most of the estimated 660 000 congenital infections resulting from these pregnancies occurred in low- and middle-income countries (LMIC) (3, 4). However, there is a trend towards increasing prevalence in high-income countries (5). Untreated maternal syphilis is associated with miscarriages, stillbirths, pre-term delivery and neonatal deaths (6).

Syphilis can infect the fetus at any stage of gestation, invoking a marked inflammatory response and widespread spirochaetal dissemination in fetal organs including the placenta (2). The risk of fetal infection is dependent on gestational age, immunological response, maternal stage of disease and, duration of appropriate maternal treatment—untreated maternal primary and secondary syphilis is associated with a high risk of infectivity (7, 8).

During uncomplicated pregnancy, the placental weight increases with gestational age but decreases in relation to fetal weight (9). Due to syphilitic effects of villitis, vascular proliferation and hypercellular villi, the placentae of neonates with congenital syphilis may be larger than expected (8, 10). In 1990, Malan et al. demonstrated a 38% frequency of heavy placentae (greater than 90th centile for weight) in congenital syphilis by plotting placental weight on a placental weight for birth weight chart based on 13,601 normal births in South Africa (11). However, there are no subsequent South African data.

South Africa is a middle-income country where syphilis prevalence is not declining as evidenced by an antenatal syphilis prevalence rate of 2% in 2015 (12). We previously described features and outcomes of 50 infants with symptomatic congenital syphilis at Groote Schuur Hospital (GSH) Neonatal Unit in Cape Town (population 4 million), South Africa (13). Although placental data were collected during this study, this was not reported, and we therefore aimed to perform a sub-study of our cohort to determine the frequency of heavy placentae.

## Methods

The index study was a retrospective record review of neonates with symptomatic congenital syphilis admitted to the GSH Neonatal Unit (NNU) from the 1st January 2011 to the 31st December 2013. The study was approved by the University of Cape Town, Health Sciences Faculty, Human Research Ethics Committee. The GSH NNU is a tertiary referral unit in Cape Town with 75 beds including 20 intensive care beds. It is the referral center for the Metro West Area of Cape Town, which has approximately forty-thousand deliveries per annum (14).

Neonates who had a positive rapid plasma reagin (RPR) with a titer >1:1 were identified from the National Health Laboratory Service database and symptomatic neonates were identified by folder review. Neonates were deemed to have symptomatic congenital syphilis if they had a positive RPR, signs which could be attributed to congenital syphilis (such as hepatosplenomegaly, rash or petechiae due to low platelets) and received penicillin as treatment. The birth weight, gestational age at birth, placental weights and neonatal outcome data were extracted. The Chi square or Fisher's exact tests were used for categorical comparisons, depending on the expected values. The student's t and Wilcoxon rank sum tests were used for comparison of parametric and non-parametric continuous variables respectively. All statistical tests were 2-sided at alpha = 0.05.

## Results

**Figure 1:**
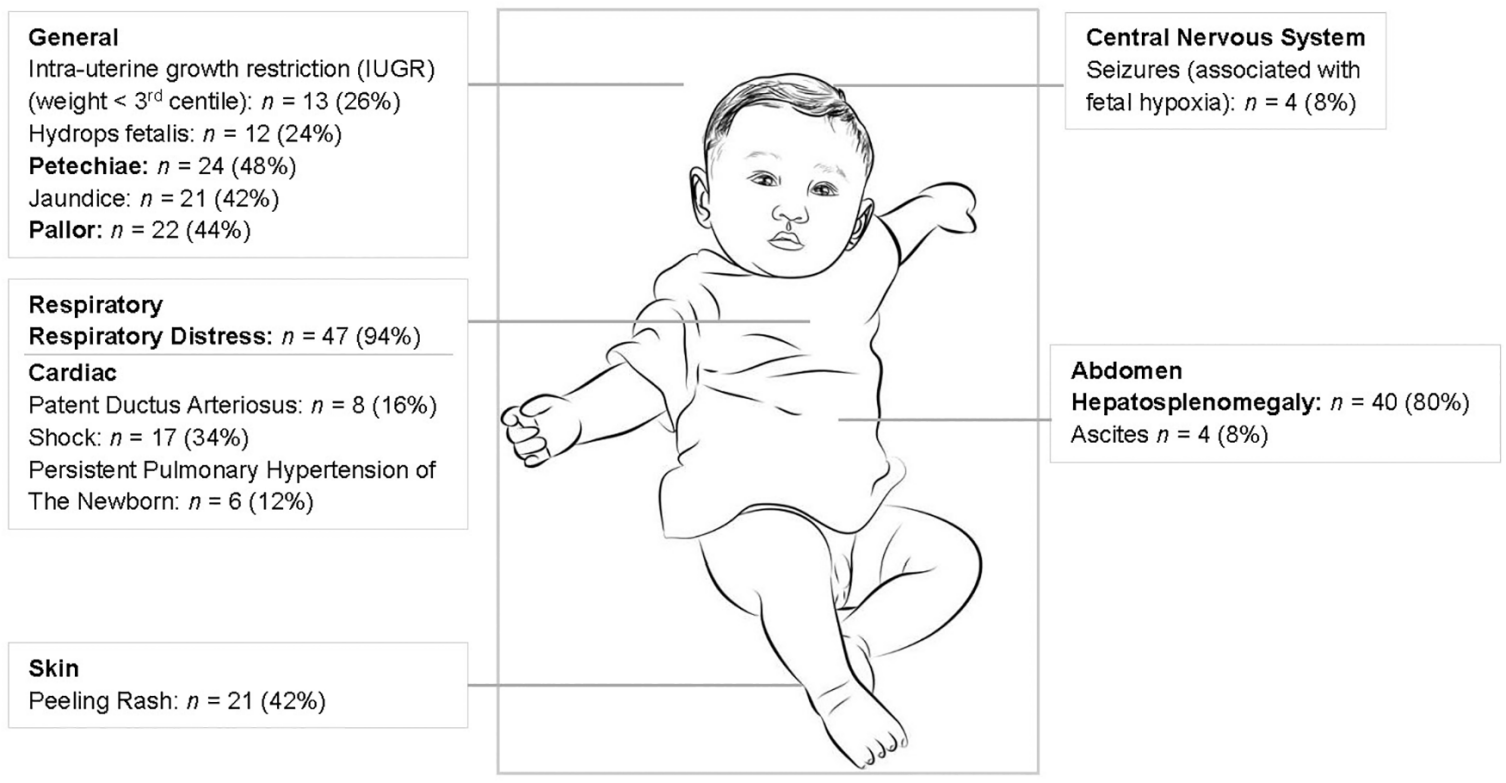
Clinical signs in the 50 symptomatic neonates.

There were 50 neonates with symptomatic congenital syphilis admitted over the three-year study period. Most (68%) of these neonates required intensive care and 19 neonates (38%) demised (13). Further clinical information is provided in Figure 1 which shows the three main signs being respiratory distress (94%), hepatosplenomegaly (80%) and petechiae (48%). Thirty-three (66%) neonates had long bone x-rays performed and 18 (55%) of these had long bone changes in keeping with congenital syphilis including metaphyseal translucent bands, erosions and periosteal reactions.

Placental weights were recorded for 37 (74%) of neonates. The weights were missing in 13 (26%) cases; three infants were born at home and the remaining missing data were related to health worker omissions. The birth weight and gestational age characteristics of the 37 neonates compared to the whole cohort are shown in Table 1; there were no significant differences between the groups—the majority were preterm (<37 weeks gestation) and low birth weight (<2,500 g).

**Table 1:** Birth weight and gestational age characteristics.

Variable	Whole cohort (*n *= 50)	Placental weight known (*n *= 37)
Birth weight *mean (SD)*	1933 (685)	1883 (669)
Low birth weight *n (%)*	39 (78%)	31 (84%)
Birth weight < 10^th^ Centile *n (%)*	13 (26%)	8 (22%)
Gestational age at birth *median (IQR)*	34 (31–37)	33 (30–36)
Preterm *n (%)*	37 (74%)	29 (78%)
Hydrops *n (%)*	14 (28)	12 (32%)

**Figure 2:**
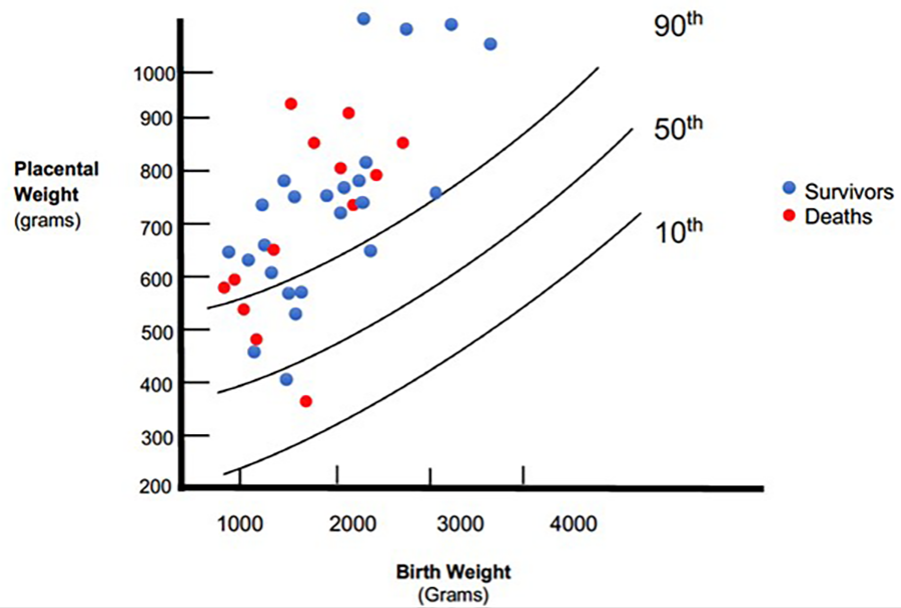
Placental weight relative to birth weight compared to population centiles.

The placental weights were plotted on a placenta for birth weight centile graph redrawn from the data published by Malan et al. (11) (Figure 2). Twenty-eight (76%) placenta weights were above the 90th centile, two (5%) were below the 50th centile and none were below the 10th centile. The frequency of heavy placentae increased as birth weight increased; all six of the neonates with a birth weight ≥2,500 g had a placenta weight above the 90th centile. Mothers had no comorbidities such as gestational hypertension that could influence placental size. Of the 12 neonates with non-immune hydrops, placentas were available for all of them. Placenta weights were above the 90th centile in 9 (75%) compared with 19 (76%) of the 25 neonates without hydrops. There was no association between death and placental weight above 90th centile (*p* = 0.691).

## Discussion

Placental weights were available for 74% of the cohort with symptomatic congenital syphilis. The majority of placentae (76%) were above the 90th centile for birth weight while the frequency of normal sized placentae decreased with increasing birth weight. There were no significant differences in birth weight and gestation between the neonates with placental weights recorded compared to the whole cohort. We did not find a universal association between hydrops fetalis and heavy placentae.

Heavy placentae occurred in our cohort at almost double the frequency compared to the 38% described in Malan's cohort, despite their similar proportions of low birth weight (83%) and preterm neonates (61%) (11). The placentae in our cohort were similarly weighed untrimmed. The difference may be related to the increased proportion of neonates with birth weight less than the 10th centile in their cohort; 41% vs. 22% (11). Although the centile charts used were from the same geographic area, they were derived 30 years previously, which may account for some of the differences.

Birth and placental weights can differ between populations, and it is important to obtain current population-specific nomograms which are not often available, especially in LMICs. Similarly, there should be standardization of placental weight/birthweight assessment—some placental centile charts include only birthweight whilst others incorporate gestational age (9). Some centers weigh the whole placenta whilst others trim the membranes and cord (15, 16). Other nomograms even attempt to adjust for factors such as maternal height and pregestational weight (17). Charts based on birth weight alone are useful in LMICs where gestational age estimation may be unreliable (18). Delayed cord clamping may also affect placental weight due to blood transfer and further data are required.

Syphilis is the most common congenital infection associated with heavy placentae but other infections such as cytomegalovirus have also been reported (19). Inclusion of placental histopathological evaluation increases the detection rate of congenital infections including syphilis where placental changes include necrotizing funisitis, villous enlargement, and acute villitis (20). Besides infections, increased placental weight/birthweight ratios have also been associated with poor perinatal outcomes (21, 22), cerebral palsy (23), hydrops fetalis, maternal diabetes mellitus and Beckwith-Weidemann syndrome (24).

The limitations with this study were the retrospective nature, missing data on 13 placentae and the lack of histological correlation. The strengths were the well described neonatal characteristics and the relatively large case series.

## Conclusions

Heavy placentae occurred in 76% of neonates with congenital syphilis. The frequency of heavy placentae increased with increasing birth weight with neonates with a birth weight ≥2,500 g all having a heavy placenta.

If placental weight is greater than the 90th centile for birth weight, neonates should be evaluated for congenital infection, hydrops fetalis and syndromes. Placental histopathological evaluation should be considered when the diagnosis is not clear.

## Data Availability

The raw data supporting the conclusions of this article will be made available by the authors, without undue reservation.
